# The Development of Inner Ear Membrane Analog for Experimental Otorhinolaryngology

**DOI:** 10.17691/stm2025.17.3.01

**Published:** 2025-06-30

**Authors:** A.A. Riabinin, O.S. Rogovaya, A.I. Kryukov, N.L. Kunelskaya, E.S. Yanyushkina, V.V. Mischenko, M.M. Ilyin, E.A. Shershunova, V.V. Voyevodin, S.V. Nebogatkin, K.I. Pomanov, E.A. Vorotelyak

**Affiliations:** Research Engineer, Laboratory of Cell Biology; Koltzov Institute of Developmental Biology of the Russian Academy of Sciences, 26 Vavilov St., Moscow, 119334, Russia; Senior Researcher, Laboratory of Cell Biology; Koltzov Institute of Developmental Biology of the Russian Academy of Sciences, 26 Vavilov St., Moscow, 119334, Russia; Professor, Corresponding Member of the Russian Academy of Sciences, Director; Sverzhevsky Research Clinical Institute of Otorhinolaryngology, Bldg. 2, 18A Zagorodnoye Shosse, Moscow, 117152, Russia; Professor, Deputy Director for Science; Sverzhevsky Research Clinical Institute of Otorhinolaryngology, Bldg. 2, 18A Zagorodnoye Shosse, Moscow, 117152, Russia; Leading Researcher, Research Department of Surdology and Pathology of the Inner Ear; Sverzhevsky Research Clinical Institute of Otorhinolaryngology, Bldg. 2, 18A Zagorodnoye Shosse, Moscow, 117152, Russia; Senior Researcher, Research Department of the Ear Microsurgery; Sverzhevsky Research Clinical Institute of Otorhinolaryngology, Bldg. 2, 18A Zagorodnoye Shosse, Moscow, 117152, Russia; Researcher, Laboratory of Stereochemistry of Sorption Processes; Nesmeyanov Institute of Organoelement Compounds of the Russian Academy of Sciences, Bldg. 1, 18A Vavilov St., Moscow, 119334, Russia; Head of the Laboratory of High Power Pulsed Technology; Institute of Electrophysics and Electric Power of the Russian Academy of Sciences, Litera A, 18 Dvortsovaya Naberezhnaya, Saint Petersburg, 191181, Russia; Senior Researcher, Laboratory of Electrodynamic Systems; Institute of Electrophysics and Electric Power of the Russian Academy of Sciences, Litera A, 18 Dvortsovaya Naberezhnaya, Saint Petersburg, 191181, Russia; Head of the Laboratory of Applied Electrophysical Research; Institute of Electrophysics and Electric Power of the Russian Academy of Sciences, Litera A, 18 Dvortsovaya Naberezhnaya, Saint Petersburg, 191181, Russia; Engineer, Laboratory of High Power Pulsed Technology; Institute of Electrophysics and Electric Power of the Russian Academy of Sciences, Litera A, 18 Dvortsovaya Naberezhnaya, Saint Petersburg, 191181, Russia; Professor, Corresponding Member of the Russian Academy of Sciences, Head of the Laboratory of Cell Biology; Koltzov Institute of Developmental Biology of the Russian Academy of Sciences, 26 Vavilov St., Moscow, 119334, Russia

**Keywords:** round window membrane, neurosensory bradyacusia, passive diffusion of dexamethasone, round window membrane model, human fibroblasts, keratinocytes

## Abstract

**The aim of the investigation** was to develop and evaluate a model of the human round window membrane (mRWM) of the inner ear that is suitable for representative studies of drug permeation and cytotoxicity.

**Biological part of the study:**

Several substrate options were tested to create the mRWM, including 2 variants of Viscoll collagen membranes (IMTEK, Russia) and a multi-component G-Derm membrane (G-DERM, Russia). In the first variant, only HaCaT epithelial cells were seeded on the membranes, and in the second variant, primary human dermal fibroblasts were seeded together with HaCaT epithelial cells (sequential application). The obtained mRWM were evaluated by morphological criteria using histochemical methods. As a result, the decision was made to use mRWM constructed on Viscoll membranes with the inclusion of both primary fibroblasts and human epithelial cells.

**Technical part of the study:**

A series of scientific experiments has been performed on the obtained mRWM aimed at studying the permeability and developing modes of electrophysical action on this biological barrier while maintaining its morphological and functional integrity and ensuring accelerated passage of dexamethasone through it. To accelerate the passage of dexamethasone across the mRWM, the electrophysical system initiated targeted iontophoresis of negatively charged dexamethasone molecules in parallel with electroporation of cell membranes in the sample. After the exposure, the residual viability of mRWM was assessed by histochemical staining with calcein and propidium iodide. The change in dexamethasone concentration after passage across the mRWM was assessed using a highly sensitive chromatograph.

**Conclusion:**

During the optimization of the mRWM fabrication protocol and the selection of suitable substrate components and cellular material, the model based on a thin Viscoll collagen membrane has been chosen as a substrate and primary human dermal fibroblasts and epithelial cells of the HaCaT line as a cellular material. The obtained experimental samples of mRWM represent a semipermeable membrane with living cells on the surface and are an alternative analog of the native structure, reproducing its geometric and morphofunctional characteristics. In addition, there has been demonstrated a method of using the for preclinical studies of electrophysical devices designed for accelerated passage of target substances through this membrane using electroporative and iontophoretic effects.

## Introduction

Hearing disorder and vestibular system impairment arising concurrently with a non-purulent pathology of the inner ear represent a serious problem due to dramatically deteriorating the quality of human life. Treatment of these diseases always includes medical therapy, where targeted transport of medicinal drugs to the focus of the pathological process is an important and difficult task. To solve the issue, efforts are directed to develop methods of targeted delivery of synthetic glucocorticoids and vascular-metabolic drugs through the biological barriers of the cochlea [1]. Application of electric field generators causing phonoelectrophoretic, electrophoretic, and iontophoretic effects is the most promising technique of drug delivery through the biological membranes of the middle ear. This approach decreases the percentage of surgical interventions and the risk of patient disability, since surgical treatment is often accompanied by high risks of hearing loss [2–4].

Implementation of the aforementioned techniques into clinical practice faces a serious problem of overcoming biological barriers (membranes) for creating sufficient therapeutic concentration in the affected area. It is known that it is possible to influence the function of the inner ear with drugs, but it is very difficult to introduce them therein [5]. The membrane formations of the inner ear hinder the penetration of the drugs due to their barrier function necessary for the protection of such a delicate apparatus as the auditory analyzer system. To overcome the biological barriers safely, it is necessary to experimentally determine the main electrophysical parameters, which impact the round window membrane (RWM) of the cochlea and the morphological capillary structure of the cochlear stria vascularis for active transport of the medicinal agent. There are two approaches to the experiments of the methods being developed: using laboratory animal models and using model systems. Various modeling techniques and their combinations to minimize usage of animals in preclinical studies have already found their application in testing novel pharmaceutical agents [6–8].

The possibility of creating tissue models, which reproduce exactly the microanatomy of the native tissue, is critically important for this technology.

Membrane formations of the inner ear (oval vestibular window and round cochlear window) refer to the natural boundary openings in the otic capsule of the temporal bone. Perilymph of the inner ear contacts indirectly with the middle ear through the membranes of these two windows. The anatomic niche of the round window opens to the scala tympani of the cochlear basal turn. The dimensions of the round window niche are highly variable. According to the data of various authors, the niche walls form a prism 1.50–2.75×1.0–2.72 mm in size, 0.65–2.12 mm deep, and an area of 2.0–3.32 mm^2^ [9–15]. RWM serves as a barrier between the cavity of the middle ear and cochlea and plays an important role in the mechanics of the middle ear and cochlea both in norm and pathology [16–19]. RWM is considered to be the main route for penetration of potentially phototoxic substances from the cavity of the middle ear to the inner ear. Additionally, RWM may be involved in the secretion and absorption of substances [20, 21]. The experiments on animals show that RWM acts as a semi-permeable membrane. Many substances with high and low molecular mass have been shown to penetrate through RWM, when they are placed in the round window niche [9]. Substances placed on RWM can pass through the cytoplasm in the form of pinocytotic vesicles or through various channels between the cells in epithelium. Cells in the connective tissue layer can phagocytize the substance and transfer it to the perilymph and/ or penetrate blood and lymphatic vessels in this layer [22]. Theoretically, once the substance reaches the perilymph, it will be directed to the spinal fluid via the cochlear aqueduct up to the scala tympani or get to the endolymph.

The accuracy and reliability of RWM model permeability have previously been studied *in vitro*. Usually, the isolated areas of the ear cochlea with the round window of some model animals served as such models: RWM of Mongolian gerbil, guinea pig, chinchilla, sheep, and others [23–26]. One of the more interesting examples of RWM shape modeling was described in the work of the Japanese researchers [27]. The human RWM shape is difficult to explore due to its location deep in the recess (in the round window niche). Nevertheless, the scientists managed to obtain the RWM sample together with the area of adjacent skull bones. Additionally, its 3D digital model was generated using laser scanning microscopy for better understanding the structure and properties of RWM.

In humans and most animals, RWM consists of three layers: the outer epithelium facing the middle ear; a layer of the connective tissue; the inner epithelium adjoining the inner ear [28]. The layer of the connective tissue contains collagen fibers as well as other elastic fibers and fibroblasts. All this ensures the main structural support to the membrane. RWM has thickenings at the edges and a thinner region closer to the center. This difference in thickness creates a small bulge in the direction of the scala tympani [10]. An average thickness of the human RWM is about 70 μm [28].

To reproduce a living RWM epithelial barrier, it would be ideal to cultivate the cells of human inner ear epithelium in combination with fibroblasts on the surface of the membrane. The role of various populations of fibroblasts in maintaining the function and structure of the collagen and epithelium has been previously studied by us [29]. However, in order to obtain the cell culture of the middle ear membrane formations, the researchers require a constant source of human biological material due to the fact that primary cultures of the human epithelial cells have a limited number of generations when cultivated *in vitro*. If the task is to develop batches of samples of round window membrane models (mRWM), there arises a need for selecting a variant of epithelial cell component, which would not only be morphologically close to the original but also readily available, not very expensive, and simple in cultivation.

**The aim of the investigation** was to develop and evaluate a model of the human round window membrane (mRWM) of the inner ear that is suitable for representative studies of drug permeation and cytotoxicity.

## Materials and Methods

### mRWM formation

The cells of the HaCaT line (100,000 per 1 cm^2^) were seeded on the substrates from Viscoll collagen (IMITEK, Russia) (samples 1 and 2) and the combination of a high density collagen and hyaluronic acid G-Derm (G-DERM, Russia) and cultivated in 5% CO_2_ conditions in the DMEM/F12 medium (PanEco, Russia) with 10% FBS, glutamine (Gibco, USA), and Normocin (InvivoGen, USA). The growth medium was changed every 3 days. 10 days after cultivation, the cells on the substrate surface generated a monolayer and mRWM was considered to be formed.

### Preparation of histological mRWM samples

10 days after epidermal cell seeding, the mRWM samples were prepared for immunostaining: they were washed three times using the phosphate-buffered saline (PBS) (PanEco, Russia), then fixed with 4% paraformaldehyde (Sigma, USA) for 30 min at room temperature. After fixation, mRWM were washed with PBS (PanEco, Russia) three times for 5 min and treated with 15 and 30% solutions of sucrose (Helicon, Russia). Next, mRWM were immersed into the commercial medium Tissue-Tek O.C.T. Compound (Sakura Finetek, Japan) for tissue freezing and were frozen in liquid nitrogen. The 7 μm mRWM cryosections were made using the Leica CM1900 cryostat (Leica Microsystems, Germany). After drying for 1 h, Mayer’s hematoxylin solution (BioVitrum, Russia) was applied on the sections (incubation for 10 min), and after washing with running water, eosin (BioVitrum, Russia) was applied (30 s incubation). After washing with distilled water, sections were embedded in the biomount (Bio Optica, Italy). The inverted fluorescent Olympus IX73 microscope with Olympus DP74 camera (Olympus, Japan) was used to visualize the samples. The images were processed in the ImageJ (https://imagej.net/) program.

### Formation of final mRWM and evaluation of permeability of different model variants for dexamethasone

In all cases, collagen membranes were placed on the inverted membrane inserts with 0.4 μm membrane pores located in the wells of the 24- well plate (SPL, China). The HaCaT cell line was applied on the surface of these membranes (100,000 cells per insert in 100 μl of media); in the variant with the intermediate layer of fibroblasts, human fibroblasts were applied first on the surface of the membrane (50,000 cells per insert in 100 μl of the medium). After 2 h, the medium was removed and HaCaT cell line was applied (100,000 cells in 100 μl of the medium). In the variant of mRWM with fibroblasts, 50,000 human fibroblasts in 200 μl of media were additionally placed on the bottom of the wells of the 24-well plate. The next day, 1 ml of the medium was poured in the well with inserts. The medium composition corresponded to that described above (see “mRWM formation”). The medium was changed every 3 days. A week after the beginning of the experiment, the inserts with mRWM samples were withdrawn from the culturing system in the 24-well plates without separation of the membrane from the insert bottom and transferred to the 96-well plates to investigate the membrane permeability. For this purpose, the lower wells of the 96-well plate were filled with PBS (300 μl per well) so that the liquid touched the membrane located on the well surface. The insert with mRWM was placed above each well so that the sample was in contact with the liquid in the well of the 96-well plate. After the dilution of dexamethasone for injection (Ellara, Russia) with a concentration of 4 mg/ml in 1:1 PBS, the obtained solution was poured into the inserts (300 μl per insert with mRWM) providing passive diffusion through mRWM, the inserts were pressed to the plate with the plate lid and left for 1 h in the incubator. In 1 h, the solution was taken from the lower wells to determine the relative concentration of dexamethasone. The experiment was repeated 3 times. The obtained numeric values allowed us to calculate the mean and standard deviation. The significance was assessed by comparing the value samples of the relative residual concentration of dexamethasone in the experimental groups and two control groups (control without the effect of electrophysical system and dexamethasone and control of the dexamethasone effect without the system).

### Experiments on speeding up the dexamethasone passage through mRWM

Ten days after seeding fibroblasts and keratinocytes, mRWM were exposed to electric current in the experimental electrophysical system developed at the Institute of Electrophysics and Electric Power of the Russian Academy of Sciences [30], which is designed for penetrating iontophoresis and electroporation of mRWM. Direct current intensity in the first experimental group was 1.0–1.3 mA and 0.5 mA in the second. Pulsed current intensity in all experiments where electroporation was used was 400 mA. Pulsed current frequency varied from 1 Hz to 10 kHz depending on the experiment (indicated further for each experiment, where pulsed current was used). Immediately after the exposure, mRWM were returned to the cultural media and placed in the CO_2_ incubator.

### Assessment of cell viability in mRWM after treatment with iontophoresis and electroporation or their combination on the sample

The viability of the control mRWM samples of various types of substrates and experimental G-Derm-based mRWM samples was studied using fluorescent dyes 20 h after the treatment. For this purpose, calcein AM (Servicebio, China) (green staining of living cells) at the 1:2000 dilution and propidium iodide (Invitrogen, USA) (red staining of nuclei in the dying cells) at the dilution of 1:3000 were introduced into the wells with the cultured samples. Staining lasted for 1 h, the medium was then replaced with a fresh one, the membranes were visualized and photographed using the Olympus IX73 inverted fluorescence microscope with Olympus DP74 camera (Olympus, Japan).

The percentage of the living and dead cells was calculated using the Image J program in manual mode (Cell counter notice plugin). Having obtained numeric values after a 3-fold repetition of the experiment, the mean and standard deviation were calculated. Calcein activity was evaluated by analyzing the intensity of green fluorescence in the field of vision.

### Determination of the dexamethasone amount passed across the RWM models

To determine the amount of dexamethasone passed through the experimental membrane samples, a highly accurate method of high-performance liquid chromatography was used. The samples, obtained after their passage through various mRWM and through the collagen membranes without cells and without dilutions, were analyzed on the Agilent 1100 liquid chromatograph (Agilent Technologies Inc., USA) using the Thermo GOLD C18 analytical column (150.0×4.6 mm, 5 μm; Thermo Fisher Scientific, USA) with 35/65 acetonitrile/ water (HPLC grade) eluent composition with 1 ml/min flow rate. The thermostat temperature was set at 25°С, UV detector wavelength at 240 nm. 1 μl of samples were introduced into chromatograph with a 5 μl syringe (Hamilton). Based on the dexamethasone signal areas obtained on the chromatograms, membrane permeability was assessed at passive diffusion comparing these areas with the area of dexamethasone signal in the initial solution.

### Statistical data processing

Normal distribution of all samples was checked using Shapiro–Wilk test in the Jamovi (v. 2.3.28) program. The results with p>0.05 values confirmed the null hypothesis on normal distribution. Statistical significance of differences in the values of dexamethasone concentrations in the solutions, as well as in the values of the measured calcein activity in the field of vision, and the number of propidium iodide-positive cells were determined using the one-way analysis of variance (ANOVA) in the Statistica 10.0 program (StatSoft, USA). The results with p<0.05 values were considered statistically significantly different from the null hypothesis. The confidence intervals in all diagrams were built based on the values of standard deviation from the sample mean.

## Results and Discussion

### Selection of the substrate for mRWM

**Figure 1.**
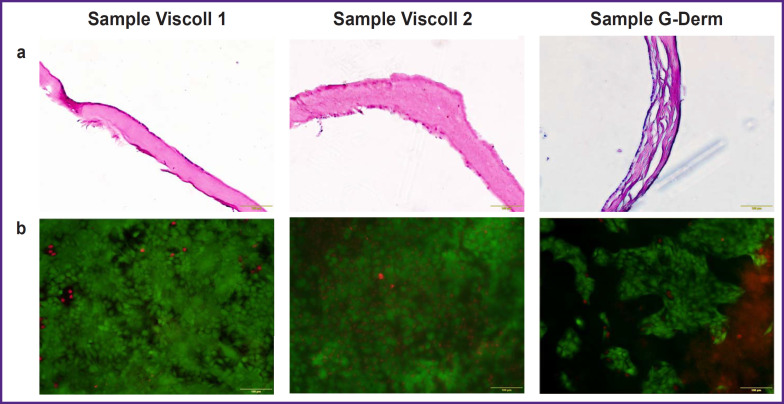
Models of the round window membrane (mRWM), 3 days after seeding the epithelial cells: (a) mRWM cross sections, staining with hematoxylin and eosin; (b) vital detection of the living and dead cells in the mRWM composition: living cells are stained with calcein (green staining), dead cells are stained with propidium iodide (red staining). Bar — 100 μm

The developed model is based on the reproduction of the main components of the human RWM: a thin collagen membrane and living epithelial cells capable of forming a uniform monolayer on its surface. Three biopolymer substrates produced in Russia were selected and tested for cytotoxicity and adhesion: two collagen Viscoll membranes fabricated from a high-density collagen, and a G-Derm biopolymer plate consisting of hyaluronic acid and collagen (Figure 1). We have tested all membranes in the cultures of human immortalized HaCaT cell line. This cell line has been found to have good adhesion to the samples and to form a relatively dense layer, however, a confluent monolayer was formed only on Viscoll membranes (Figure 1 (b)).

In samples from Viscoll, the structure on the section looks more uniform, in G-Derm sample, the structure contains greater number of layers, cavities, and irregularities (Figure 1 (a)). Such a rough laminated membrane structure is likely to possess increased permeability for dexamethasone, which will distort the experimental data at the next stages of the study. The test on viability of the epidermal cells based on vital staining with propidium iodide and calcein has shown high viability of the cellular component in mRWM in all experimental groups (see Figure 1 (b)). Besides, the membrane thickness on the section was found not to exceed 200 μm in all tested samples and is comparable with a human RWM thickness. Thus, it has been decided to use collagen Viscoll membranes as a basic matrix, since the morphology and density of the collagen fiber bundles in this type of the carrier are similar to those in RWM of cats, guinea pigs, and sheep, which have been described in the literature [21, 26]. It is known that fibroblasts are present in the connective tissue of the round window of the human inner ear, which may be a neglected factor in estimation of the permeability of the round window for a target substance — dexamethasone [10]. According to the literature data, various populations of fibroblasts influence differently both the condition of the collagen matrix and higher indicators of the epithelial cell survival, therefore it was decided to prepare several mRWM variants based on the total fraction of fibroblasts with diverse variants of epithelialmesenchymal interaction of two cell types [29, 31, 32].

### Assessment of passive permeability of various mRWM for dexamethasone

**Figure 2.**
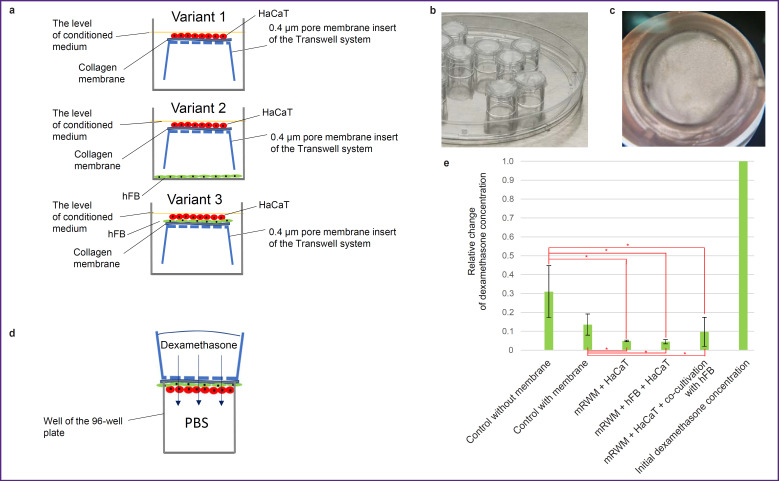
Assessment of permeability of the round window membrane models (mRWM) for dexamethasone: (a) schematic illustrations of different mRWM variants used in the experiment; hFB — human fibroblasts; (b) collagen membranes located on the inserts of the Transwell system; (c) photos of mRWM after a week of cultivation; (d) a diagram of testing permeability of the obtained mRWM for dexamethasone; PBS — phosphate-buffered saline; (e) a histogram demonstrating residual dexamethasone concentration after passing across mRWM by passive diffusion. All samples used for building the histogram were tested for normality; * statistical significance of the ANOVA results is at the level of p<0.05; confidence intervals reflect standard deviation from the mean

To assess dexamethasone permeability, 3 variants of mRWM have been formed in the system with membrane inserts (Figure 2 (a)). Once a cell layer has been formed on the membranes (Figure 2 (c)), mRWM were considered completed and the experiment on the diffusion of dexamethasone solution at a concentration of 2 mg/ml was started as described in the section “Material and Methods” (Figure 2 (d)). After the sample collection and analysis, it has been found that all mRWM variants were semipermeable for dexamethasone, however, they function as barriers and let only part of the substance pass through: dexamethasone was detected in all samples but its concentration decreased significantly. The final dexamethasone concentration was 13.5% in the model with one collagen membrane without cell material; 5.0% in mRWM with epithelial cells only; 4.5% in mRWM with human fibroblasts (hFB) and epithelial cells; 9.7% in mRWM with epithelial cells and co-cultivation with hFB (Figure 2 (e)). Thus, the resulting concentration of dexamethasone decreased by 10–20 times after passing through various types of mRWM relative to the initial one. According to the literature data, similar tests were not conducted on analogous round window models of the human middle ear. In this connection, we compared the results of measuring passive diffusion of dexamethasone at 20 mg/ml concentration through our mRWM and RWM taken from the ear of guinea pigs [33]. 60 min after the introduction of dexamethasone, the diffusion rate in our model was at least 10 times higher and closest in the variant of mRWM with hFB and HaCaT. The difference in the rate of passive diffusion may be caused by the differences in the extracellular matrix in the matrix composition.

Summarizing the obtained data, we may conclude that the epithelial layer being formed by the cells of the HaCaT line ensures a reliable decrease of collagen membrane permeability. Introduction of an additional cell type into the system only insignificantly enhances these properties. The impact of fibroblasts on the survival of the epithelial layer after electrophysical exposure needs further additional investigations.

**Figure 3.**
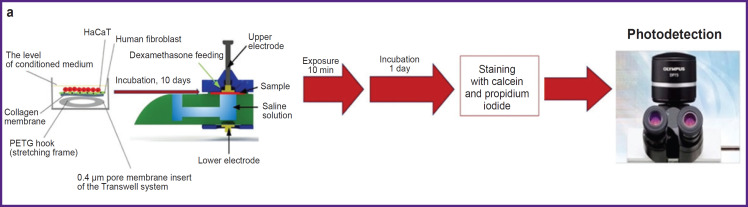

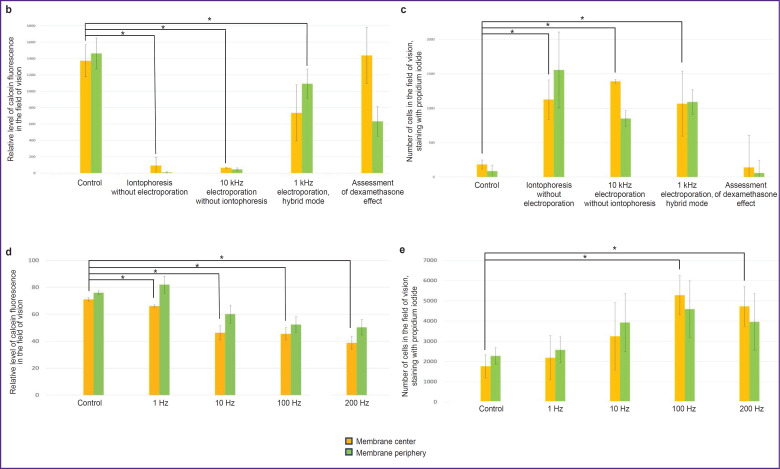
Testing passive and active diffusion of dexamethasone through the models of the round window membranes (mRWM): (a) schematic diagram of the experiment; (b) and (c) relative viability of the cells in the field of vision (calcein activity) and the number of dead cells in mRWM (staining with propidium iodide), respectively: in the control group before the exposure; in the group with dexamethasone before the exposure (evaluation of dexamethasone effect); in the experimental groups — after the exposure to the direct current without electroporation (iontophoresis without electroporation at DC voltage), after electroporation at 10 kHz pulse frequency (electroporation without iontophoresis) and after exposure to direct current voltage in combination with pulsed voltage with 1 kHz pulse frequency (hybrid mode); (d) and (e) relative viability in the field of vision (calcein activity) and the number of dead cells in mRWM (staining with propidium iodide), respectively: in the control group before the exposure and in the experimental groups after the exposure to electroporation with 1, 10, 100, 200 Hz pulse frequency; (b)–(e) all samples used for building the histogram were tested for normality; * statistical significance of the ANOVA results is at the level of p<0.05; confidence intervals reflect standard deviation from the mean

At the next stage of the study, the obtained mRWM were used for control trials of the experimental electrophysical system [30] (Figure 3 (a)), which was designed to accelerate the process of passing dexamethasone through the membrane being the model of the round window of the inner ear.

In the process of testing the experimental system in various modes it has been determined that only the mode with direct or 10 kHz pulsed current greatly reduces cell viability relative to the control. However, the viability of the cell material decreases to a lesser extent if the direct and 1 kHz pulsed currents are used in combination: about 2 times in the center of the membrane (in the area of direct contact of the membrane with the electrode) and by a third at the periphery (in the region without contact). Besides, while assessing cytotoxicity of pure dexamethasone without exposure to the system, it has been estimated that viability values were closer to the group with combined impact, where a lower current was applied (Figure 3 (b)). It is worth noting that the number of dead cells was sufficiently great in all operating modes and exceeded the control group by 10 times. The difference between the groups “iontophoresis without electroporation”, “10 kHz electroporation without iontophoresis”, and “1 kHz” was statistically insignificant. The quantity of dead cells in the groups of the assessment of dexamethasone effect and control did not differ (Figure 3 (c)).

Testing the combined operating mode of the system with active electroporation and iontophoresis and a lower pulsed current frequency (within 1 to 10 Hz), it has been found that cell viability dropped insignificantly but no reliable difference in the number of dead cells between these two groups was observed (Figure 3 (d), (e)). At the same time, a greater frequency of the pulsed current (over 100 Hz) results in a significant decrease of cell viability (up to 2.5 times relative to the control) (see Figure 3 (d), (e)). It may occur due to the osmotic pressure impairment in cells and strong sample heating at a higher pulse frequency. A large mRWM viability after the exposure may be due to a lower current (1 and 10 Hz). In the process of a long cultivation of mRWM after the exposure to the system with the pulsed current frequency of 1 and 10 Hz, colonies of keratinocytes and fibroblasts were detected around the membranes on the bottom surface of the 6-well plates, in which mRWM were cultured. It indirectly confirms good residual viability of the cells in mRWM and their migration and proliferation potential after exposure to the system at these modes.

## Conclusion

In the process of our investigation, we managed to create the mRWM of the human inner ear representative by its parameters and to develop the method of assessing the viability of the cell material in the model after the impact of the studied factor. It is especially crucial, since a reliable evaluation of the native RWM viability is impossible due to the problems of its extraction without damage. The developed model may be used to test new methods of drug delivery to the endolymph and perilymph of the inner ear. In particular, it has been shown in the present work that mRWM demonstrates a satisfactory permeability for a target substance — dexamethasone in the conditions of passive diffusion. The optimal operating parameters of the previously developed system (pulsed current frequency) have also been determined, ensuring viability preservation and accelerated passing of dexamethasone across mRWM by means of iontophoresis and electroporation. This model including human fibroblasts and keratinocytes on a dense semipermeable matrix may find further application in fundamental research and as a test system for pharmaceutical developments, since it facilitates native interactions between epithelial and mesenchymal cell fractions and stimulates matrix normalization with the cells included in its composition.
